# Long-term spaceflight composite stress induces depression and cognitive impairment in astronauts—insights from neuroplasticity

**DOI:** 10.1038/s41398-023-02638-5

**Published:** 2023-11-08

**Authors:** Yishu Yin, Junlian Liu, Quanchun Fan, Shuang Zhao, Xiaorui Wu, Jiaping Wang, Yu Liu, Yongzhi Li, Weihong Lu

**Affiliations:** 1 School of Medicine and Health, Harbin Institute of Technology, Harbin, 150001 China; 2 School of Chemistry and Chemical Engineering, Harbin Institute of Technology, Harbin, 150001 China; 3 National and Local Joint Engineering Laboratory for Synthesis, Transformation and Separation of Extreme Environmental Nutrients, Harbin, 150001 China; 4 China Astronaut Research and Training Center, Beijing, 100094 China; 5 The Intelligent Equipment Research Center for the Exploitation of Characteristic Food & Medicine Resources, Chongqing Research Institute, Harbin Institute of Technology, Chongqing, 401135 China

**Keywords:** Molecular neuroscience, Depression

## Abstract

The environment on the space station is quite unique compared to Earth, which is a composite of multiple stressors, such as microgravity, isolation, confinement, noise, circadian rhythm disturbance, and so on. During prolonged space missions, astronauts have to stay in such extreme environments for long periods, which could induce adverse effects on both their physical and mental health. In some circumstances, this kind of long-term spaceflight composite stress (LSCS) could also induce depression and cognitive impairment in various ways, including dysregulating the neuroplasticity of the brains of astronauts, which should be attached to great importance. Here, we have comprehensively reviewed the impact of individual and combined stressors on depression and cognitive function during long-term spaceflight, explained the underlying mechanisms of those effects from the perspective of neuroplasticity, and current countermeasures for mitigating these challenges. This review provides insights into LSCS and potential neuroplasticity mechanisms, current with potentially great impact for understanding and mitigating the mental health risks and traumas of career astronauts and space tourists.

## Introduction

**Fig. 1:**
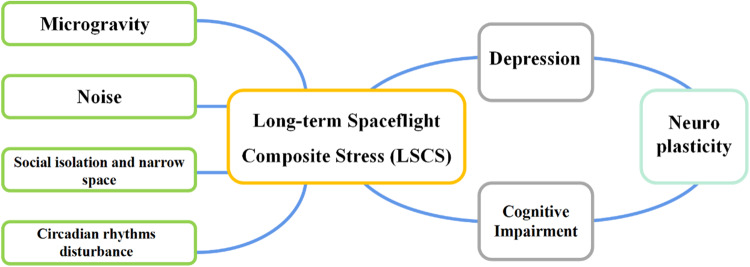
**Long-term spaceflight composite stress**. Long-term spaceflight composite stress mainly includes microgravity, isolation, confinement, noise, circadian rhythm disturbance, and so on, which could induce depression and cognitive impairment through various ways. Here, we have comprehensively reviewed the impact of individual and combined stressors on depression and cognitive function during long-term spaceflight, and explained the underlying mechanisms of those effects from the perspective of neuroplasticity.

Human beings are conducting more and more space missions in the space station, which is just the first step to exploring the vast universe. And maybe in the near future, we will achieve more ambitious goals, such as the Mars expedition [1]. During the spaceflight, the most critical decisive factor should be the astronauts themselves. Whether they can maintain a high level of performance efficiency determines the success and safety of long-duration space missions. However, astronauts will face various stressors in the space station, including microgravity, isolation, confinement, noise, circadian rhythm disturbance, and so on [2]. The complicated space station environment will not only affect their physical functions but may also induce psychological problems, such as anxiety, depression, and cognitive decline. The National Aeronautics and Space Administration (NASA) found that 22.8% of male and 85.2% of female astronauts had symptoms of anxiety, while 34.8% of male and 43.2% of female astronauts had symptoms of depression. Moreover, the average annual incidence of severe mental and psychological disorders in long-term spaceflights exceeding 600 days exceeds 60% [3]. Furthermore, almost all astronauts returning from space missions have reported cognitive and operational issues related to the central nervous system (CNS) [4]. The famous “NASA TWINS STUDY” also provided a scientific finding that extended mission duration (12 months) may negatively affect postflight cognitive performance for up to six months [5]. The investigation of spaceflight impact on CNS has practical implications, while most are still remaining unclear up to date though there is an increasing interest in it [6], of which neuroplasticity deserves particular attention. Neuroplasticity concerns the adaptive ability of the brain and neurons when coming to new stimuli or environments [7] and maybe a new insight for us to understand the neurobiological basis of depression and alterations in cognition during long-term spaceflight. This paper, therefore, reviews stressors alone and in combination that may have detrimental impact on the astronaut’s emotion and cognition in the space station during long-term spaceflight and the potential neurological basis of those effects from the perspective of neuroplasticity as well as effective countermeasures for cognition and depression challenges (Fig. 1). Since the radiation dose is relatively small in the space station which is in the near-Earth orbit and is protected by geomagnetic field [8], we would not take the radiation effects into consideration this time.

## Effects of long-term spaceflight stressors on depression and cognitive function

### Microgravity

Microgravity is one of the most important stressors that distinguishes space stations from the ground, by which the vast majority of physical, chemical, and biological phenomena taking place on Earth are governed. The physical and biological adaptations observed during space missions reveal the crucial role of gravity in the evolutionary development of humans as well as a potential link between microgravity and the onset of diseases [9]. In space, microgravity will result in adverse effects on the human body, including on the cardiovascular system [10], musculoskeletal [11], respiratory, and CNS [12], especially on the emotional and cognitive functions [13]. One study showed that after 6-month exposure to microgravity on the International Space Station (ISS), eight astronauts showed significant deficits in cognitive functions, such as manual dexterity, dual tasking, motion perception, and even a significantly reduced ability to operate the vehicle when compared to the ground-based controls [14]. Apart from the distinctive research environment provided by the ISS, parabolic flight, dry immersion, and “head-down bed rest” (HDBR) methods are widely used to investigate microgravity on Earth. Evidence showed that simulated microgravity could also exhibit negative effects on human emotions and cognitive functions, mainly in the form of a decrease in positive emotions, abnormal mood swings of fear and anxiety, and a decrement in task performance. Chen et al. found the subjects’ short-term memory was impaired, while the depression and anxiety feelings were not significantly different after the 45-day −6°HDBR [15]. Rodent studies also demonstrated that simulated microgravity would induce anxiety and depression [16], along with cognitive changes [17].

However, there exists some debate. Wollseiffen and his crews suspected cognitive impairment was a composite of complex spaceflight stressors instead of microgravity itself, and they found neuro-cognitive performance can even be enhanced during short periods of microgravity [18]. Different studies have selected different tasks to measure alterations in cognitive levels, so they may not be strongly comparable. Though these results cannot be attributed only to exposure to microgravity, they still hold significant implications for future investigations concerning the adaptation of neuroplasticity under long-term spaceflight.

### Noise

The unique space station environment is full of different levels of noise produced by the onboard equipment, including fans, exercise equipment, environmental control, and life support equipment, as well as payloads [19]. Crewmembers have to be exposed to the noise over the course of their stay in the space station, which may induce health risks, such as reductions in hearing sensitivity, disruptions of crew sleep, interference with voice communications and crew task performance, and reduced alarm audibility [20]. It has been reported that the monotony of noise can degrade habitability in space stations, especially with inadequate rest during long-duration missions [21]. It is noticeable that long-term exposure to noise can also cause depression and anxiety. Nonetheless, the effect of noise associated with the space station is less investigated, so we select ground-based cases here to illustrate. As we know, transportation noise is a common type of noise that is associated with depression and anxiety. Studies have shown that individuals who are exposed to the disruptive effects of traffic noise are at an elevated risk of experiencing depressive symptoms [22]. Long-term exposure to noise seems to be most detrimental, mainly via noise annoyance [23], which may trigger negative emotions and activate the pressure response of the hypothalamic-pituitary-adrenal (HPA) axis that is involved in the pathophysiology of depression [24]. Sleep disorders caused by noise are also related to the occurrence of depression [25]. Furthermore, rodent studies suggested that rats exposed to noise for a long period would induce depression-like behaviors [26]. In terms of cognitive impairment, evidence indicated that noise has adverse effects on children’s learning, short-term memory, reading and writing abilities, and long-term exposure to noise may affect their cognitive development imperceptibly [27]. A study investigating traffic police in high-traffic areas and office staff in quiet areas found that workers exposed to noise became more susceptible to stressors in neurophysiology and behavior, which may be related to emotional status and cognition [28].

In many cases, it is difficult to set noise as the only variable, so there still exists some debate about whether the pathological changes are caused by noise [29]. Moreover, noise level and noise annoyance may jointly and independently influence the risk of depression [23]. Actually, non-auditory effects of noise on human health, such as sleep disturbance, mental health, physiological function, depression, and cognitive impairments, are the results of noise as a general stressor instead of sound energy itself [30] and long-term exposure to it could have a detrimental influence on the body potentially. Thus, it is reasonable to speculate that the presence of noise within the confines of the space station may potentially result in depressive symptoms and cognitive deficits for astronauts.

### Social isolation and confinement

The emotional and cognitive performance of individuals can be impacted by stress arising from confinement and social isolation, particularly astronauts on long-duration missions. They will face long distances away from Earth, long separation from family and friends, cultural issues and interpersonal stressors, narrow and crowded environments, and the resulting homesickness. Those factors can be totally summarized as social isolation and narrow space issues, which are huge emotional pressures and may be one of the most severe stressors [31]. As social beings, humans experience significant decrements in their cognitive and affective states when isolated from others, inducing loneliness, anxiety, paranoia, and depression [32]. Research findings have indicated that long-term social isolation can lead to conflict and emotional deterioration, which may, in turn, lead to problematic behaviors that interfere with productivity and interpersonal relationships [33]. However, the real data from the space station is not nearly enough, so several types of analogs are used to study the potential impacts of social isolation and confinement, such as submarines, polar stations, purpose-built simulated space capsules, and other isolated and confined extreme (ICE) environments [34]. The results of the MARS105 simulation study showed that increased feelings of loneliness and abandonment may interfere with cognitive performance, especially the performance cost of switching from one task to another [35]. Results from the MARS500 experiment showed subjects experienced depression and mental decline after a long period of isolation [36]. Moreover, this circumstance is equally applicable to the crews of submarines. The incidence of anxiety and depression has significantly increased in crew members from both surface and underwater long voyages, according to the self-rating anxiety scale (SAS) and self-rating depression scale (SDS). Their concentration, reaction time, and memory were also affected. It should be acknowledged that a significant proportion of these effects exhibit a linear dose–response relationship, wherein the extent of behavioral and cognitive impacts increases with prolonged exposure to ICE environments [37].

### Circadian rhythm disturbance

The space station is generally located at an altitude of 330–480 km and takes approximately 90 min to orbit the Earth each time [38], which means there are 16 sunrises and sunsets within a 24-h period, resulting in a significant departure from the familiar 24-h diurnal cycle experienced on Earth. Thus, circadian rhythm disturbance is a common phenomenon during spaceflight and is considered as a critical risk factor during long-term missions by NASA [39]. Any natural light that astronauts are exposed to in the space station may affect their circadian rhythm adaptation [40]. Although the disturbed rhythm can be adjusted by light control to some extent, it may not counter the impact of solar and moon gravitational rhythm changes totally. Additionally, another significant contributor to circadian rhythm disturbance is the huge workload of astronauts during space missions, which makes them stay concentrated for a long time to complete tasks [41].

Circadian rhythm disturbance may result in serious impacts on astronauts, including sleep loss, decrements in performance, loss of concentration and memory, impaired alertness, as well as depression and anxiety [42]. Research indicated that disturbance of the circadian rhythm system can lead to neurobiological dysfunction, which may manifest as symptoms of depression [43]. A previous study suggested that altered circadian rhythms were a causal factor in the development of mood disorders, and a shared aspect of these mechanisms was observed among them. [44]. Naismith asked the shift workers with circadian rhythm disorders to fill out the “Depression Self-Assessment Form” and found that the degree of depression of the subjects was directly related to circadian rhythm disorders [45]. Besides, the resulting sleep loss can also lead to cognitive, motor, and neurobehavioral deficits [46].

### Composite environmental stressors

Understanding the multifaceted impact of the space environment on the human body from a singular perspective is one-sided, for stressors rarely act alone but rather in concert [47]. Consequently, it is imperative to thoroughly investigate the alterations induced by the collective environmental stressors. Unfortunately, little information is currently available on how the combined stressors influence the human body. Our investigation found that only a limited pool of researchers has conducted such experiments on rodents. Ma et al. initiated a study in which they simulated the space station environment through tail-suspension, isolation, and circadian rhythm disturbance for 28 days and found that the space composite environment model can cause severe damage to the cognitive function of rats [48]. In another study, Wang et al. used a combination of tail suspension, noise, narrow space, and circadian rhythm disturbance to simulate the complex space environment and found that depression-like changes appeared in the mood of rats under this circumstance. Additionally, the influence of complex environments on the brain nerves of model rats was more serious than single factors, such as microgravity. This tail-suspension compound restraint model can cause depression-like behaviors in rats, and a decrease in the ability to form reward conditioned reflexes at the same time, which in turn leads to cognitive function damage in the rats. Wu and her colleagues performed a study in which they used four factors of tail-suspension, noise, isolation, and circadian rhythm disturbance to simulate the long-term space composite stress environment and found that the model rats have depression-like behaviors and neuronal damage in the hippocampus region [16].

The examination of combined stressors remains in a nascent stage. This information gap has significantly hindered us to realistically evaluating spaceflight stressor risks to human deep space exploration, which requires us to invest more energy in the future.

## Neuroplasticity and its alterations in response to spaceflight

Neuroplasticity underlies a range of changes that occur within the human neurological system in spaceflight, encompassing physical, mental, and behavioral changes [49], and can be considered as synaptic plasticity at the cellular level and alterations of neural networks at the system level [50]. The former can be manifested as structural plasticity as well as functional plasticity, which is essential for learning and memory processes [51]. Commonly used techniques in the study of neuroplasticity include electroencephalography (EEG), structural and functional magnetic resonance imaging (MRI), Functional MRI, and transcranial magnetic stimulation (TMS) [7]. The most commonly used technique for spaceflight is EEG due to its notable advantages of portability and user-friendly. In EEG, multiple electrodes are placed along the scalp to monitor and measure the electrical activity of the brain, which has been employed many times in the ISS. However, the low spatial resolution (5–9 cm) of EEG makes it difficult to attribute findings to precise regions [52]. MRI, a state-of-the-art neuroimaging technique with high spatial resolution, allows for extracting functional, structural, and biochemical information from precise brain regions and is an excellent tool for dynamically tracking brain neuroplasticity responses in vivo [53]. Functional MRI is an MRI-based technique that has quite a high spatial resolution (<1 cm) and is commonly used to study activity patterns and functional connectivity of brain regions. TMS is a painless, non-invasive brain stimulation technique that can be deployed to investigate the brain’s response to space missions by collecting data on cortical excitability, neuroplasticity, and brain connectivity levels [54]. These methodologies collectively contribute to a deeper comprehension of neuroplasticity.

Generally, neuroplasticity dysregulation is mainly manifested in the following ways, including changes in the structure of cerebral tissues, reduced neuronal regeneration or apoptosis, impaired signal transduction pathways, and impaired synaptic plasticity. So how is neuroplasticity altered or disrupted in spaceflight? In a study of an astronaut on 169-day long-duration ISS missions, researchers identified that prolonged spaceflight would cause alterations in his brain function through fMRI investigations, including decreased connectivity of key parts of the vestibular system and between the left cerebellum and right motor cortex, which may explain the reduced vestibular function and motor control abilities [55]. In another MRI study, Vincent and coworkers compared pre- and post-flight MRIs of 27 astronauts to evaluate the effects of spaceflight on human brain structure, which is also the first report on changes in human brain structure with spaceflight. Their results showed a significant reduction in extensive volumetric gray matter, while small but localized gray matter increases in sensorimotor brain regions, reflecting that brain plasticity changes in response to spaceflight and different neuroplastic processes could take place simultaneously [56]. Moreover, Roberts and colleagues compared brain images of 18 astronauts before and after long-term missions by MRI and found that narrowing of the central sulcus, upward shift of the brain, and narrowing of the cerebrospinal fluid spaces at the vertex occurred frequently [57]. Their group has previously analyzed MRIs of the changes in the brains of eight healthy volunteers undergoing a long-term HDBR. Their results indicated that the brain as a whole can move within the skull in response to gravity changes, and these locally occurring morphologic changes may lead to possible brain function changes [58]. Concerning HDBR, Zhou and colleagues performed a study on 16 healthy male volunteers and investigated whether the functional architecture of their brains was altered after 45 days of HDBR. The results demonstrated that simulated microgravity specifically disrupted functional networks anchored in the left anterior insula and the anterior part of the middle cingulate cortex, which specifically respond to the degree of subjective salience, including cognitive and emotional, which may account for the impairments in cognitive function that occur during spaceflight [59]. Cassady et al. also initiated a spaceflight analog study in which they investigated changes in brain connectivity during 70 days of HDBR. Different from the above studies, their study observed the temporal dynamics of brain connectivity by selecting 7-time points, rather than selecting two-time points, before and after spaceflight. They found significant changes in the functional connectivity of vestibular, somatosensory, and motor networks. Furthermore, they also found that functional connectivity alterations were significantly associated with changes in sensorimotor and spatial working memory performance, suggesting that neuroplasticity may contribute to adaptation to the environment [60].

Based on the aforementioned research, it has been observed that the brain undergoes morphological and functional connection changes as a result of exposure to the space environment and its analogs, which are considered alterations of brain neuroplasticity. It is noteworthy that these alterations occasionally exhibited dysfunctionality while at other times demonstrated adaptability [61]. Given that the real data from space are very scarce and the sample size is quite small, as well as ground analogs is difficult to substitute the unique space environment, whether these two seemingly conflicting patterns of brain changes happen at the same time remains unsolved.

## Potential neuroplasticity basis for LSCS-induced depression and cognitive deficits

The intricate causative mechanisms of LSCS-induced depression and cognitive impairment among astronauts remain elusive and inadequately comprehended, which encompass multiple aspects, including the reduction of neurotransmitter levels, HPA axis dysfunction, oxidative stress and inflammatory responses, and so on. It is well understood that the environment within the space station, where astronauts reside and conduct space missions for extended durations, is characterized as extremely stressful. Increasing evidence showed that neuroplasticity would be disrupted under chronic stress, and then depression and cognitive impairment would be precipitated and exacerbated [62]. Thus, we suspect that astronauts are prone to neuroplasticity disruption and further develop depression and cognitive impairment under LSCS. Our inclination towards this supposition is grounded in the following evidence: (1) Hippocampal alterations. The hippocampus can be negatively impacted by various spaceflight stressors. It may be particularly affected by microgravity, as hippocampal CA1 neurons appear to be more sensitive to the effects of microgravity than other rough surface neurons, exhibiting decreases in area, perimeter, and synaptic cleft [63]. Some evidence suggests that social isolation and confinement lead to hippocampal dysfunction, including shrinkage of CA1 and reduced contextual fear conditioning, which is associated with a reduction in markers of synaptic plasticity in this region [64]. In addition, LSCS-induced sleep loss reduces connections between the hippocampus and frontal and parietal regions, which results in memory impairment [65]. It also reduces protein synthesis in the hippocampus, closely linked to neuroplasticity, and impairs hippocampal neurogenesis [66]. The alteration observed in the hippocampus is a manifestation of neuroplasticity, which is directly connected to the regulation of emotions and cognitive function. Consequently, any modifications in this region are bound to impact depression and cognitive deficits. (2) Neural regulator alterations. Long-term spaceflight has a significant impact on the fundamental regulators of brain neuroplasticity, specifically neurotransmitters such as 5-HT and DA, as well as neurotrophic factors like CDNF and GDNF. At the same time, these regulators have clearly emerged as targets in the pathogenesis of depression [67–69]. Further, the deficiency of BDNF is considered to play an important role in the pathogenesis of depressive disorders. While there is currently no clear evidence showing that BDNF was affected remarkably by spaceflight, one possible explanation is that down and/or upregulation of BDNF in response to spaceflight may not be a long-term phenomenon, which might take place during the early exposure [70]. (3) Interactions among spaceflight stressors. Some compounds of cellular, molecular, and neurochemical indicators may occur due to the interplay of spaceflight stressors. Additionally, certain stressors may mutually influence one another or give rise to secondary consequences, which may further intensify the behavioral and neurological impacts. In such cases, depression and cognitive impairment may arise as secondary outcomes.

## Countermeasures for long-duration space missions

In light of the previously discussed stressors encountered during spaceflight in orbit, the question arises as to what action can be undertaken to address these challenges. Currently, there exist several interventions that approach the reduction of adverse effects on cognition and emotion, including psychological methods, ergonomic methods, physiological methods, and so on [71].

Kanas came up with three domains of countermeasures, namely pre-flight preparation, in-flight support, and post-flight readaptation [72]. The selection of crewmembers and their pre-flight training are widely recognized as the most commonly employed pre-flight countermeasures [47]. In candidates’ selection, the formulation of the selection strategy is very important. Not only are selection criteria developed based on specific tasks, but a comprehensive assessment, including performance tests, personality questionnaires, biographical data analysis, interviews, and behavioral observations, should be undertaken. At the same time, communication skills, interpersonal skills, and intercultural skills are also indispensable. In addition, candidates with low neuroticism and high agreeableness, conscientiousness, openness, and extroversion, may lead to better team dynamics [73], while those with a history of mental illness and mental illness tendencies should be eliminated in time. Psychological training should be intensive and high-intensity before a mission so that the number of unforeseen contingencies would be reduced and the crew remains calm in highly stressful situations. Emotional training should also be performed to improve the crew’s emotional intelligence (EI) and their ability to manage and regulate their emotions. Evidence shows that emotional training helps to reduce negative emotional stress in highly uncertain situations [74]. Furthermore, the crew should be trained together on the ground in order to have strong cohesion when performing tasks.

In-flight countermeasures include monitoring and supporting the well-being of the astronauts, which can be both provided onboard and remotely. Remote monitoring and ground support staff are important monitoring methods to detect early emotional changes in astronauts. They need to make judgments and provide appropriate advice and guidance at the first time. Astronauts may sometimes be reluctant to mention emotional stress, which requires us to use both subjective reports and non-invasive methods such as EEG monitoring, audio monitoring, video behavioral observation, etc., to assess their emotions and cognition. Additionally, self-testing of astronauts themselves plays an essential role, which requires adequate training in advance. Moreover, medical kits should be provided onboard. It is difficult to completely avoid anxiety, depression, or sleep problems, so artificially restoring the crew’s hormonal balance may be more helpful in strengthening their emotional regulation. For instance, alendronate and testosterone are used to increase cognitive activity and regulate emotional alterations in an HDBR study [75]. However, some drugs may have serious side effects, and some are addictive, so it’s better to choose non-invasive methods.

Personalized leisure-time activities should be strengthened to mitigate monotony and loneliness, and corresponding support activities should be provided to different astronauts according to their characteristics and preferences. They are allowed to carry personal items that meet defined size and weight requirements. Additional leisure material can also be provided to orbital crews via resupply flights as needed. Spacecraft habitat design is an ergonomic intervention that aims to give astronauts a better habitation experience and reduce the adverse impact of isolation and confinement on their mood, which is one of the key ways to solve the problem of monotony and loneliness. Burattini and colleagues have come up with a novel design for habitation to satisfy dimensional, comfort, and psychological needs [76]. Other important support activities include private psychological sessions, informal ground contacts in space and news from the Earth, and regular opportunities to stay in close contact with family and friends on the Earth. Supporting the astronaut families during a mission helps keep them focused on mission goals and lessens their concerns about possible family issues and responsibilities [31].

Due to the complexity of psychological problems, space psychology experts have also developed some new psychological support means. One of the most promising new psychological support methods may be based on virtual reality (VR) technology, which can present astronauts with images of Earth’s nature and daily life, preventing sensory deprivation and monotony, as well as negative emotions caused by homesickness. In addition, the utilization of voice assistants, social robots, and spacecraft greenhouses also contributes to cultivating positive psychological effects for astronauts [77].

Post-mission readaptation involves individual issues and family issues. Some astronauts will find themselves thrust into the spotlight and social media after their return, as well as integration difficulties due to prolonged separation from their families. These issues can be dealt with by supportive debriefings and formal counseling resources, protected privacy during the readjustment period, and a period of time to reconnect to get used to living on Earth again [72].

## Conclusion

During long-term space missions on the space station, astronauts will encounter various stressors and multiple stresses, including microgravity, noise, circadian rhythm disturbance, isolation and confinement, etc. The integration of these factors will contribute to the development of composite stress, resulting not only in physical and mental harm to astronauts but also potentially leading to cognitive impairment and depression. Although we understand that the LSCS will be a major obstacle against sustained human space missions, including the exploration of Mars, prior academic work has neglected to explore solutions to cope with it, lacking in-depth investigation and systematic interventions. In this review, the impact of stressors alone and in combination on depression and cognition impairment were discussed, and the possible neurobiological basis of the development of which are explored from the perspective of neuroplasticity. Finally, interventions and possible mitigation strategies are listed. However, neuroplasticity-induced depression and cognition impairment by LSCS are discussed only as a highly plausible theoretical speculation without sufficient evidence. There is still an urgent need for broader research to increase the impact of spaceflight on the human brain, and expanding this knowledge is critical to ensuring the safety and efficiency of future space missions.

## Data Availability

The data that support the findings of this study are available from the corresponding author upon reasonable request.
